# Drp1 depletion protects against ferroptotic cell death by preserving mitochondrial integrity and redox homeostasis

**DOI:** 10.1038/s41419-024-07015-8

**Published:** 2024-08-27

**Authors:** Stephan Tang, Anneke Fuß, Zohreh Fattahi, Carsten Culmsee

**Affiliations:** 1 Institute for Pharmacology and Clinical Pharmacy, Philipps-University Marburg, Marburg, Germany; 2 Marburg Center of Mind, Brain and Behavior—CMBB, Marburg, Germany; 3 Institute of Reconstructive Neurobiology, Neurodevelopmental Genetics, University Bonn, LIFE & BRAIN Center, Bonn, Germany

**Keywords:** Cell death in the nervous system, Stress and resilience, Cell death, Energy metabolism

## Abstract

Mitochondria are highly dynamic organelles which undergo constant fusion and fission as part of the mitochondrial quality control. In genetic diseases and age-related neurodegenerative disorders, altered mitochondrial fission-fusion dynamics have been linked to impaired mitochondrial quality control, disrupted organelle integrity and function, thereby promoting neural dysfunction and death. The key enzyme regulating mitochondrial fission is the GTPase Dynamin-related Protein 1 (Drp1), which is also considered as a key player in mitochondrial pathways of regulated cell death. In particular, increasing evidence suggests a role for impaired mitochondrial dynamics and integrity in ferroptosis, which is an iron-dependent oxidative cell death pathway with relevance in neurodegeneration. In this study, we demonstrate that CRISPR/Cas9-mediated genetic depletion of Drp1 exerted protective effects against oxidative cell death by ferroptosis through preserved mitochondrial integrity and maintained redox homeostasis. Knockout of Drp1 resulted in mitochondrial elongation, attenuated ferroptosis-mediated impairment of mitochondrial membrane potential, and stabilized iron trafficking and intracellular iron storage. In addition, Drp1 deficiency exerted metabolic effects, with reduced basal and maximal mitochondrial respiration and a metabolic shift towards glycolysis. These metabolic effects further alleviated the mitochondrial contribution to detrimental ROS production thereby significantly enhancing neural cell resilience against ferroptosis. Taken together, this study highlights the key role of Drp1 in mitochondrial pathways of ferroptosis and expose the regulator of mitochondrial dynamics as a potential therapeutic target in neurological diseases involving oxidative dysregulation.

## Introduction

Mitochondria are the key organelles of energy metabolism maintaining cellular bioenergetics through aerobic oxidative phosphorylation, particularly in neurons with their constant high energy demand. In addition, mitochondria are highly dynamic organelles that also play essential roles in redox signaling, calcium buffering, iron-sulfur cluster biosynthesis, and programmed cell death. Mitochondrial dynamics involve the ability of the organelles to modulate their size, shape and distribution within the cell, and this is coordinated by fission and fusion events. In neurons, balanced mitochondrial dynamics are crucial to fulfill the high energy demand, axonal transport and calcium homeostasis, which is critical for synaptic transmission, plasticity and cell survival [1, 2]. Consequently, imbalanced mitochondrial fission-fusion dynamics in neurons can contribute to neuronal dysfunction and, thereby, to various neurodevelopmental and age-related neurodegenerative disorders [3, 4].

Mitochondria undergo subdivision during the fission process, which is responsible for the subcellular distribution of mitochondria under physiological conditions, for cytochrome C release during apoptosis e.g., in response to oxidative stress, and for mitochondrial quality control through facilitating mitophagy of damaged organelles [5, 6]. The key player in executing mitochondrial fission is Dynamin-related protein 1 (Drp1). Drp1 is a GTPase that induces mitochondrial constriction at fission sites. Upon activation through phosphorylation at Ser616 and dephosphorylation at Ser637, Drp1 translocates from the cytosol to the mitochondrial outer membrane, where it is recruited by several adaptor proteins like MiD49, MiD51, MFF, or FIS1. Drp1 then assembles into oligomeric ring-like structures around the mitochondrial outer membrane to divide the mitochondria at these fission sites [7–9].

Mutations in the *DNM1L* gene encoding Drp1 result in severe and predominantly lethal encephalopathy known as EMPF1 (MIM#614388). Affected patients exhibit an elongated and tubular mitochondrial network in their fibroblast cells, caused by decreased mitochondrial fission [10–12]. On the other hand, Drp1 hyperactivity, e.g., in conditions of lethal damage upon hypoxia or GSH depletion, can lead to excessive mitochondrial fragmentation and organelle dysfunction. Such extensive mitochondrial fission was also associated with the production of reactive oxygen species (ROS) by the damaged mitochondria and amplified oxidative stress-related neuronal cell death [13, 14]. Similar phenomena have also been observed in neurodegenerative disorders like Alzheimer’s disease, where the Drp1 inhibition was shown to alleviate the pathology [15–18]. Drp1 double knockout mouse models are lethal, emphasizing its key role in cell survival. In contrast, the heterozygote mice with partial reduction in Drp1, however, show significantly reduced levels of free radicals and lipid peroxidation [17, 19, 20]. In line with this, a role for Drp1 was implicated in neuronal cell death in response to glutamate-induced oxidative cell death and Drp1 silencing or pharmacological inhibition prevented mitochondrial fission, loss of mitochondrial membrane potential, and oxidative cell death [14, 16].

Ferroptosis is an iron-dependent form of oxidative cell death that is more and more frequently linked to regulated cell death underlying the progression of various different pathological processes including neurodegenerative diseases [21, 22]. Ferroptosis is described as a regulated necrotic cell death pathway, comprising excessive iron accumulation that leads to lipid peroxidation in the cell membrane, subsequent membrane rupture, and ultimately cell death [23]. The imbalanced intracellular redox homeostasis in ferroptosis is closely tied to mitochondria; the primary source of highly regulated and balanced intracellular ROS production. This highlights the importance of mitochondrial quality control to keep the redox balance thereby protecting cells from oxidative damage, which may otherwise amplify key mechanisms of ferroptosis [24]. However, the specific impact of Drp1 in mitochondrial quality control and particularly the fission process on cellular integrity in the context of ferroptosis remains elusive.

In this study, we aimed to provide insights into the role of Drp1 as a key factor in mitochondrial fission and how its inhibition influences the resilience against ferroptosis in neuronal cells. Therefore, CRISPR/Cas9-based genome editing was applied for generating stable and specific Drp1 knockout in immortalized mouse hippocampal HT22 neurons. Following this, we investigated how preventing mitochondrial fragmentation contributed to preserving mitochondrial integrity and the mitochondrial and cellular redox state in the context of ferroptosis induced by erastin and RSL3.

## Results

### Knockout of Drp1 prevents ferroptosis-mediated mitochondrial fragmentation

**Fig. 1:**
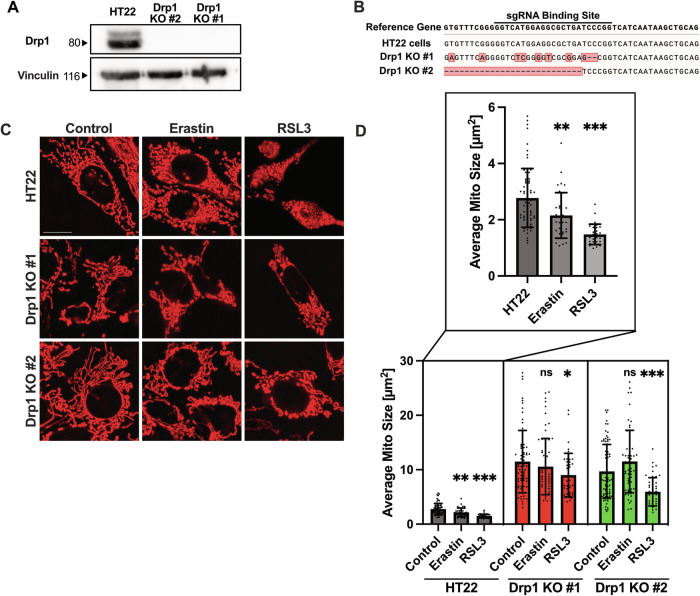
Knockout of Drp1 abolishes ferroptosis-mediated excessive mitochondrial fragmentation in mouse hippocampal HT22 cells. **A** Western Blot of HT22 cell lysates, which were transfected with or without a plasmid containing a single guide RNA targeting Drp1, the Cas9 nuclease, and an eGFP reporter gene following clonal selection. Antibodies targeting Drp1 and Vinculin as loading control shows complete knockout of Drp1 in two colonies. **B** Sequencing results of the amplified region containing the sgRNA Binding Site at the genome encoding the Drp1 protein. The sequencing results of respective cell lines are depicted against the reference gene, showing deletions and changes in nucleotides in the Drp1 KO cell lines. **C, D** Drp1 KO leads to elongated mitochondria. The cell lines were treated with 0.5 µM erastin and 100 nM RSL3 respectively for 6 h and stained with MitoTracker Deep Red to analyze mitochondrial morphology. Mitochondria of the treated HT22 cells are heavily fragmented. Mitochondria of the Drp1 KO cell lines are enlarged and less fragmented after ferroptosis induction in comparison to the HT22 controls. **C** Representative pictures of respective cell lines are depicted. Scale bar 20 µm. **D** Values are shown as mean ± SD including the single values; *n* = 29-80 replicates. ns non-significant, *p < 0.05 compared to untreated control, **p < 0.01 compared to untreated control, ***p < 0.001 compared to untreated control (One Way ANOVA, Bonferroni’s post-hoc Test).

To investigate the role of Drp1 in ferroptosis, mouse hippocampal HT22 cells were transiently transfected with a plasmid containing the Cas9 nuclease and a single guide RNA targeting the GTPase domain of Drp1. Western blot analyses of the generated HT22 colonies showed a successful knockout of Drp1 protein abundance (Fig. 1A). The region encompassing the sgRNA binding site was amplified using the genomic DNA extracted from the generated colonies and then sequenced, which showed successful cleavage of the sgRNA binding site in the generated Drp1 KO colonies (Fig. 1B).

It was previously shown that during oxidative death, mitochondria are heavily fragmented [14, 16, 25]. To expand our previous findings on effects of Drp1 deficiency, we treated HT22 and Drp1 KO cells with the established ferroptosis inducing compounds erastin and RSL3 (Fig. 1C, D). The analysis of mitochondrial morphology revealed fragmented mitochondria within 6 h of ferroptosis induction (Fig. 1D) and elongated mitochondria as a result of Drp1 knockout in HT22 cells, thereby validating the loss of Drp1 functionality in the knockout cell lines (Fig. 1C). Knockout of Drp1 abolished erastin-mediated mitochondrial fragmentation (*P* < 0.001 Drp1 Knockouts in comparison to HT22 cells) (Fig. 1D). Notably, mitochondria of the RSL3-treated cells in the Drp1 KO cell lines were more fragmented in comparison to the Drp1 KO controls (*P* = 0.041 in Drp1 KO #1 and *P* < 0.001 in Drp1 KO #2), however, overall size of the mitochondria were highly elongated in comparison to the wildtype HT22 cells (*P* < 0.001 RSL3-treated Drp1 KO cells in comparison to RSL3-treated HT22). These results showed that Drp1 deletion resulted in elongated networks of mitochondria in the neuronal cells. Furthermore, the pronounced fragmentation of mitochondria in erastin-mediated ferroptosis, was prevented in cells with genetic Drp1 deletion.

### Knockout of Drp1 reduces ferroptosis-mediated mitochondrial redox signaling

Ferroptosis is defined as an iron-dependent cell death pathway [23]. In order to examine mitochondrial contribution in ferroptosis, we studied the trafficking of iron during Drp1 deficiency in conditions of erastin and RSL3 treatment. We assessed cytosolic iron content by staining the cells with PhenGreen SK Diacetate, which is quenched upon iron binding. The basal iron content in the Drp1 KO #1 cell line was significantly lower than in the HT22 controls, which was not significant in the Drp1 KO #2 line. Strikingly, incubation with a ferroptosis inducer led to increased uptake of iron into the cytosol in Drp1 KO lines between 2 to 6 h, but not in HT22 controls (Fig. 1SA, B). After 8 h of erastin and RSL3 treatment, cytosolic iron levels decreased significantly in comparison to the basal iron level in all cell lines. RSL3 treatment did not significantly influence cytosolic iron levels between 2 and 8 h of treatment in HT22 controls. In Drp1 KO cell lines, however, iron levels in the cytosol increased between 2 and 6 h of RSL3 treatment and decreased after 8 h of incubation.

**Fig. 2:**
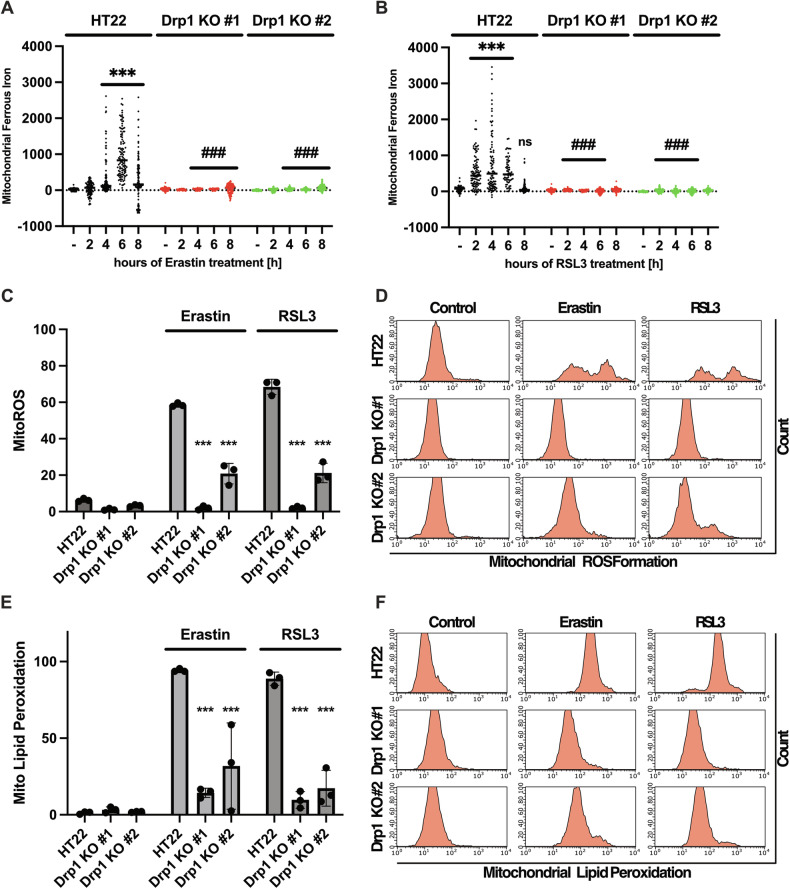
Inhibition of Drp1 stabilizes ferroptosis-mediated trafficking of ferrous iron and redox state. Knockout of Drp1 abrogates ferroptosis mediated excessive mitochondrial uptake of iron. Mitochondrial iron uptake was assessed by staining the cells with Mito-Ferro Green. The cells were treated with 0.5 µM erastin (**A**) and 100 nM RSL3 (**B**) for the indicated time points. The individual values are depicted as an arbitrary fluorescence value; *n* = 100-125 replicates; ns non-significant to respective untreated control, ***p < 0.001 compared to respective untreated control, ### *p* < 0.001 in comparison to the respective time-point of the treated control (One Way ANOVA, Bonferroni’s post-hoc Test). **C** Knockout of Drp1 abrogates erastin and RSL3 mediated excessive mitochondrial ROS formation. The cells were treated with 0.5 µM erastin and 100 nM RSL3 for 16 h and stained with the red fluorescent dye MitoSOX following flow cytometry. The values are depicted as percent of gated MitoSOX positive cells. Values are depicted as mean ± SD; 5000 cells per replicate of *n* = 3 replicates. ***p < 0.001 compared to treated control (One Way ANOVA, Bonferroni’s post-hoc Test). Representative blots are shown in (**D,** **E**) Drp1 KO protects against erastin and RSL3 mediated mitochondrial lipid peroxidation. The cells were stained with MitoPerOx fluorescent dye after treatment with 0.5 µM erastin and 100 nM RSL3 for 16 h, following flow cytometry. The percent of gated MitoPerOx positive cells was quantified. Values are projected as mean ± SD; 5000 cells per replicate of *n* = 3 replicates. ***p < 0.001 compared to treated control (One Way ANOVA, Bonferroni’s post-hoc Test). Representative blots are shown in (**F**).

We also measured the mitochondrial iron content using the green fluorescent dye Mito-FerroGreen. Analysis of the HT22 control cells undergoing ferroptosis revealed that mitochondrial iron levels reached a peak at 6 h of erastin and at 4 h of RSL3-treatment and decreased again after 8 h of treatment time. Strikingly, the knockout of Drp1 completely abolished the mitochondrial uptake of iron (Fig. 2A, B) between 4 to 8 h of erastin treatment and 2 to 6 h during RSL3 incubation. As a positive control, the HT22 control cells were co-treated with the iron chelator Deferoxamine (DFO), which has been established to inhibit cytosolic ROS production by chelating iron [23]. Our results showed that co-treatment with DFO inhibited the uptake of iron into mitochondria in both conditions of erastin and RSL3-induced ferroptosis (Fig. 1SC).

High levels of iron in mitochondria contribute to the generation of mitochondrial ROS [26]. Therefore, we assessed mitochondrial superoxide formation by performing flow cytometric measurements with MitoSOX. Erastin and RSL3 treatments induced excessive formation of MitoROS, while knockout of Drp1 inhibited this effect (Fig. 2C, D). Notably, Drp1 KO #2 did not completely abrogate mitochondrial ROS formation in comparison to Drp1 KO #1.

Recent studies suggested that lipid peroxidation occurred in mitochondria because of the aforementioned increases in MitoROS formation [27]. Consequently, we stained the cells with MitoPerOx, which specifically binds to peroxidized lipids inside the mitochondria. In line with the results from MitoSOX measurements, mitochondrial lipid peroxidation was highly elevated during ferroptosis, which was abrogated in the Drp1 KO cells (Fig. 2E, F).

These results suggest that the knockout of Drp1 significantly downregulated mitochondrial redox signaling through inhibition of mitochondrial iron uptake and subsequent mitochondrial (lipid) ROS production.

### Knockout of Drp1 impedes ferroptotic impairment of mitochondria and stabilizes ferroptosis-mediated decline of cellular and mitochondrial bioenergetics

**Fig. 3:**
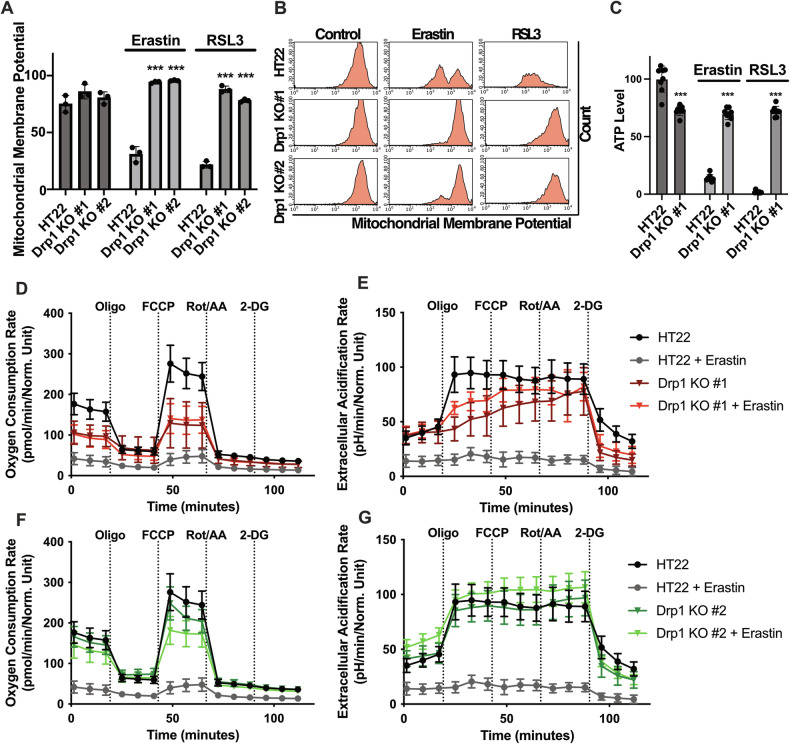
Drp1 KO impedes ferroptosis-mediated impairment of mitochondria by decreasing mitochondrial respiration. **A** Drp1 KO protects against erastin and RSL3 mediated loss of mitochondrial membrane potential. The cells were treated with 0.5 µM erastin and 100 nM RSL3 and stained with TMRE following flow cytometry. The number of cells with TMRE fluorescence were gated and quantified. Values are shown as mean ± SD; 5000 cells per replicate of *n* = 3 replicates. ***p < 0.001 compared to treated control (One Way ANOVA, Bonferroni’s post-hoc Test). Representative blots are shown in (**B**). **C** The cell lines were seeded into 96-well plates at a density of 7000 cells/well to measure ATP levels after the cells were subjected to ferroptosis using 0.5 µM erastin or 100 nM RSL3 for 16 h. Values are depicted as mean ± SD of *n* = 8 replicates; ***p < 0.001 compared to untreated control (One Way ANOVA, Bonferroni’s post-hoc Test). **D**–**G** The oxygen consumption rate and the extracellular acidification rate was measured using the Seahorse XF96-Analyzer. The cells were treated with 0.5 µM erastin for 16 h and measured. The oxygen consumption rate and the extracellular acidification rate were normalized to respective protein content. The traces are depicted as mean ± SD at *n* = 6–8.

During ferroptosis, mitochondrial integrity is impaired through the damage caused by the elevated ROS state [14, 25]. Former studies have shown that with the decrease of mitochondrial respiration [28] and the stabilization of mitochondrial ROS production [14], mitochondrial integrity was preserved, as determined by measuring the mitochondrial membrane potential (MMP). Therefore, the mitochondria were stained with TMRE, which is a red fluorescent cationic dye that binds to the net negative charge of the inner mitochondrial membrane and is used for measuring alterations of the MMP. Our results showed that ferroptosis-mediated loss of mitochondrial membrane potential was abolished by the genetic depletion of Drp1 (Fig. 3A, B).

Since mitochondrial integrity was preserved in the Drp1 KO cells upon ferroptosis induction, we addressed mitochondrial function and cellular bioenergetics. Assessment of overall ATP levels revealed preserved ATP production in the Drp1 KO #1 cell line with both ferroptosis inducers (Fig. 3C). Further, oxygen consumption rate (OCR) as a parameter for mitochondrial respiration was quantified using the Seahorse XF Analyzer. The OCR measurement in the Drp1 KO cell lines in comparison to the HT22 controls revealed a significant reduction of basal respiration as well as maximal respiration after injection of FCCP in the Drp1 KO #1 cell line compared to HT22 controls, but not in the Drp1 KO #2 cell line (Fig. 3D, F). Further, the glycolytic reserve after oligomycin-injection was attenuated under basal conditions in the Drp1 KO #1 cell line in comparison to the wildtype controls, which again was not detected in the Drp1 KO #2 cell line (Fig. 3E, G). After 16 h of erastin incubation, complete loss of oxidative phosphorylation and glycolytic activity was preserved by knockout of Drp1 (Fig. 3D-G). Strikingly, Drp1 depletion did not protect against RSL3-mediated decline of mitochondrial respiration and only the Drp1 KO #1 cell line maintained glycolysis after RSL3-induced ferroptosis (Fig. 2SA–D).

Overall, Drp1 knockout preserved mitochondrial integrity and energy supply in conditions of ferroptosis. These protective effects were more pronounced in the Drp1 KO #1 cell line which also exerted reduced mitochondrial respiration under basal conditions and showed rescued glycolytic activity after induction of ferroptosis.

### Knockout of Drp1 preserves cellular integrity independent of lipid peroxidation and GPX4 downregulation

One biochemical hallmark of ferroptosis is lipid peroxidation-mediated rupture of the cellular membrane. One key protein in neutralizing hydroperoxides that cause lipid peroxidation is Glutathione Peroxidase 4 (GPX4) [29], which is directly inhibited by RSL3 or indirectly inhibited by erastin via depletion of glutathione.

**Fig. 4:**
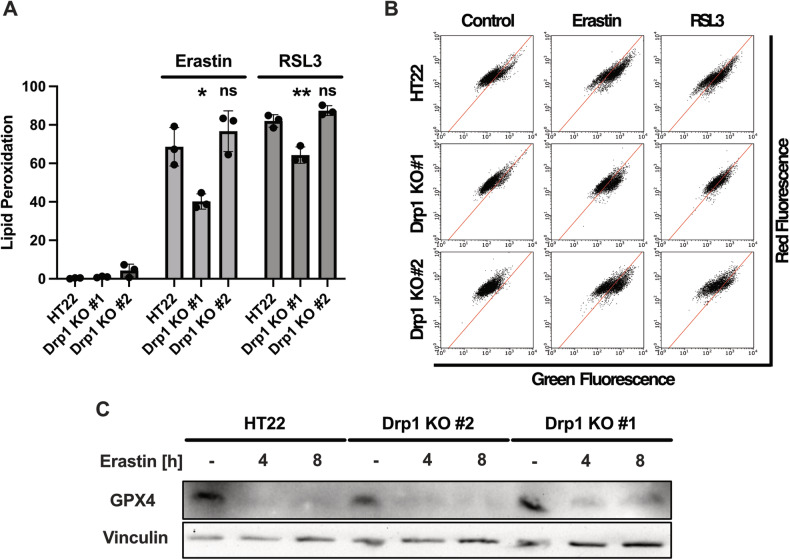
Drp1 KO-mediated protection against ferroptotic impairment of mitochondria is independent of lipid peroxidation. **A** Knockout of Drp1 fails to prevent erastin and RSL3 mediated lipid peroxidation. Lipid peroxidation was measured using BODIPY-C11 using flow cytometry. The cells were treated with 0.5 µM erastin and 100 nM RSL3 for 8 h. Values are shown as mean ± SD; 5000 cells per replicate of *n* = 3 replicates. ns non-significant, *p < 0.05, **p < 0.01 compared to treated control (One Way ANOVA, Bonferroni’s post-hoc Test). Representative blots are shown in **B**. **C** Western Blot analysis of cell lysates that were subjected with 0.5 µM erastin treatment for the indicated time points. Antibodies against GPX4 and Vinculin as loading control show decline of GPX4 protein abundancy in all cell lines.

We assessed lipid peroxidation by staining the cells with BODIPY-C11 followed by flow cytometry. Strikingly, our results showed that Drp1 knockout attenuated the erastin and RSL-3 mediated lipid peroxidation in the Drp1 KO #1 cell line, while no significant change was detected in Drp1 KO #2 cell line (Fig. 4A, B). However, lipid peroxidation in both cell lines significantly increased during ferroptosis in comparison to the untreated controls.

Also, since GPX4 plays a major role as defense mechanism against ferroptosis, the cells were treated with erastin in order to deplete glutathione synthesis. Western Blot analysis of the treated cells showed that GPX4 protein abundance declined in HT22 as well as Drp1 KO cells (Fig. 4C), suggesting that the protection against mitochondrial hallmarks of ferroptosis was independent of GPX4 activity, and solely mediated by Drp1 deficiency in the knock out cell lines. Overall, these findings suggest that genetic depletion of Drp1 significantly protected against ferroptotic stress by preserving mitochondrial integrity independently of accumulation of lipid peroxidation or GPX4 regulation.

### Drp1 deficiency is responsible for increased cellular resilience against ferroptosis

**Fig. 5:**
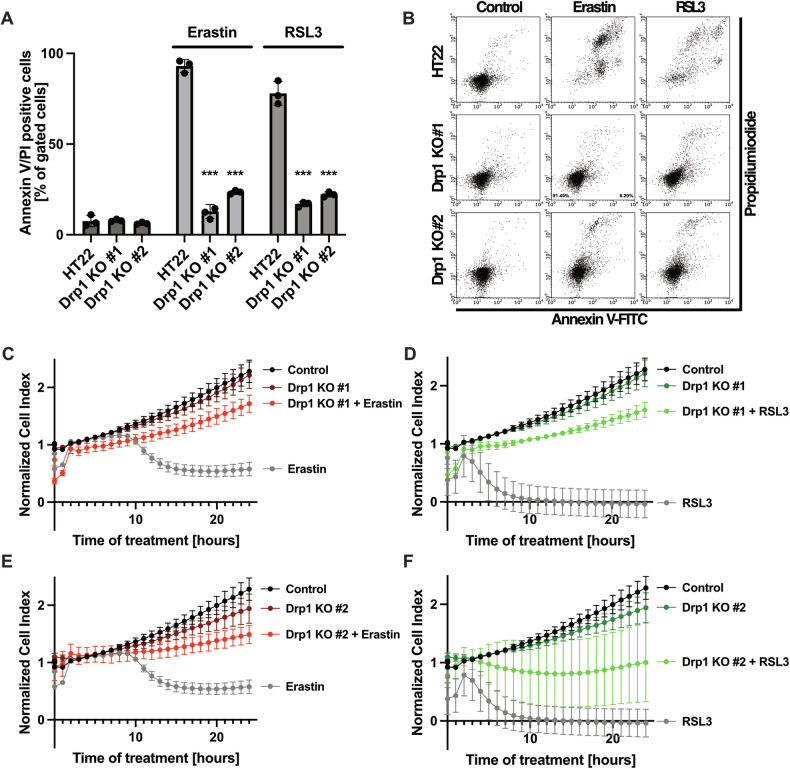
Drp1 Knockout prevents ferroptotic cell death. **A, B** Drp1 knockout protects against ferroptotic cell death. The cells were treated with 0.5 µM erastin and 100 nM RSL3 for 16 h and stained with Annexin V-FITC and propidiumiodide following flow cytometry. Representative blots are shown in (**B**). Values are shown as mean ± SD; 5000 cells per replicate of *n* = 3 replicates. ***p < 0.001 compared to treated control (One Way ANOVA, Bonferroni’s post-hoc Test). **C**–**F** Real-time impedance measurements show erastin mediated cell death at 10 h of treatment and RSL3 mediated cell death at 4 h of treatment, that was abolished by the knockout of Drp1. xCELLigence measurements were conducted during the treatment of 0.5 µM erastin and 100 nM RSL3. The cell index is normalized to the timepoint of treatment. Values are shown as mean ± SD.

Ultimately, we tested for overall cell viability through flow cytometric measurements with Annexin V-FITC and propidiumiodide staining (Fig. 5A, B) and real-time impedance measurements (Fig. 5C–F). These experiments confirmed that the knockout of Drp1 completely abrogated ferroptotic cell death induced by erastin or RSL3.

**Fig. 6:**
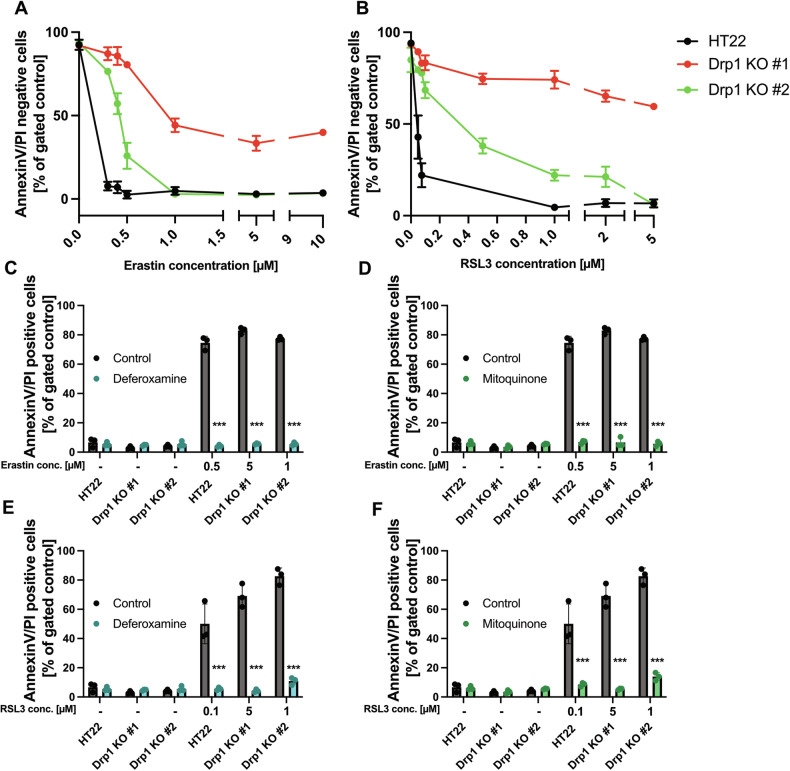
Chelation of iron and scavenging of mitochondrial ROS provide protection additionally to Drp1 desensitization towards ferroptosis. All cell lines were treated with increasing concentrations of erastin (**A**) and RSL3 (**B**), following flow cytometric measurements after staining with Annexin V/Propidiumiodide. Values are shown as mean ± SD; 5000 cells per replicate of *n* = 3 replicates. The cell lines were subjected to ferroptosis, using the IC_100_ concentrations of erastin (**C, D**) and RSL3 (**E, F**) for 16 h, during the cotreatment of 10 µM Deferoxamine (**C, E**) and 0.5 µM Mitoquinone (**D, F**) and measured using flow cytometry after staining with Annexin V/Propidiumiodide. Values are shown as mean ± SD; 5000 cells per replicate of *n* = 3 replicates. ns non-significant, ***p < 0.001 compared to treated control (One Way ANOVA, Bonferroni’s post-hoc Test).

Further, we evaluated the protective capacity of the Drp1 KO cell lines against increasing exposure to ferroptotic stimuli. For this, we titrated increasing erastin and RSL3 concentrations in the HT22 wildtype cells and in the Drp1 KO cell lines (Fig. 6A, B). The results confirmed that the Drp1 KO #1 cell line were more resistant to higher concentrations of the ferroptosis inducers than the Drp1 KO #2 cell line. However, cell viability of both Drp1 KO cell lines eventually declined at the highest concentrations of the applied ferroptosis inducers. This effect was revertible by co-treating the cells with the iron chelator Deferoxamine and the mitoROS scavenger Mitoquinone (MitoQ), which exerted additional protective capabilities against ferroptosis through iron chelation and mitochondrial ROS scavenging, respectively (Fig. 6C–F).

**Fig. 7:**
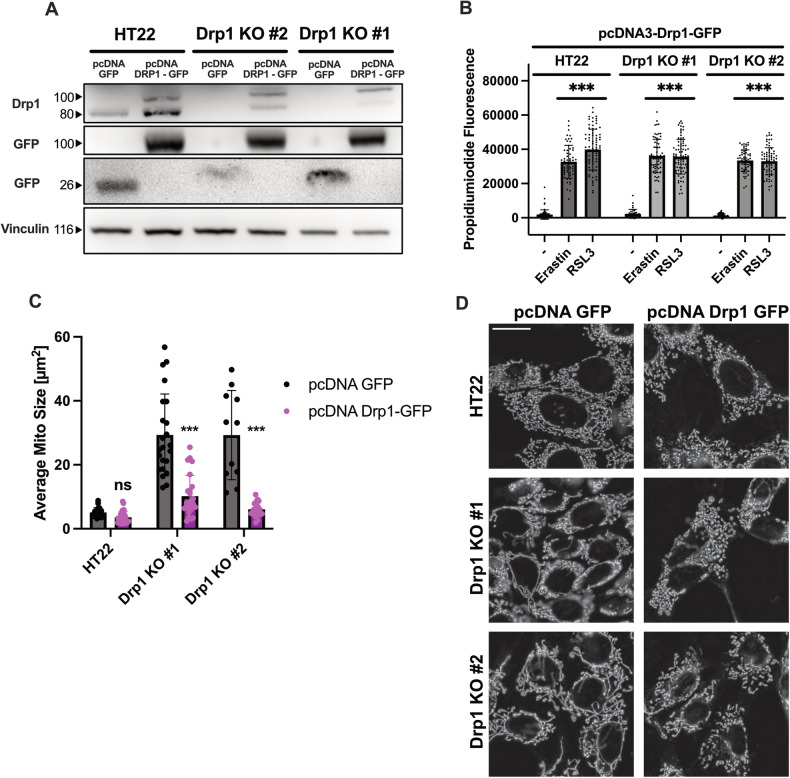
Retransfection of Drp1 re-sensitizes CRISPR/Cas9 Drp1 knockout cells towards ferroptosis. **A** Western Blot analysis of cell lines, that were transfected with a pcDNA3-GFP control or a pcDNA3-Drp1-GFP plasmid for 24 h. Antibodies against Drp1, GFP and Vinculin as loading control were used, showing successful transfection of the proteins. **B** Transfection of Drp1 re-sensitizes the cells towards erastin and RSL3 mediated ferroptosis. Only cells, that were GFP positive were used for the quantification, measuring propidiumiodide fluorescence after 0.5 µM erastin and 100 nM RSL3 treatment for 16 h as an arbitrary fluorescence value. Values are shown as mean ± SD; *n* = 40-80 cells. ***p < 0.001 compared to untreated control (One Way ANOVA, Bonferroni’s post-hoc Test). **C** The cell lines were transfected with either the Drp1-GFP vector or the control, showing fragmentation of the mitochondria with transfection of Drp1. Values are shown as mean ± SD of *n* = 11-27 replicates. ns non-significant, ***p < 0.001 compared to untreated control (One Way ANOVA, Bonferroni’s post-hoc Test). Representative pictures are shown in (**D**). Scale bar 20 µm.

Finally, in order to validate that the protection against ferroptosis was indeed mediated by the Drp1 knock out, rescue experiments were conducted using a plasmid containing the Drp1 protein coupled to a GFP reporter gene that was transfected into the cells. The results revealed restored sensitivity of the Drp1 KO cell lines towards ferroptosis as shown by propidiumiodide staining (Fig. 7A, B) and re-fragmentation of the mitochondria in Drp1 KO cell lines (Fig. 7C, D).

Overall, these results demonstrated that Drp1 deficiency significantly contributed to the resilience against ferroptotic cell death, and this protective effect was further accelerated through iron chelation and radical scavenging.

## Discussion

The present study demonstrates that the knockout of Drp1 enhances cellular resilience against ferroptotic cell death by attenuating ferroptosis-mediated accumulation of mitochondrial ROS production and through rescue of mitochondrial membrane integrity. Interestingly, Drp1-deletion also prevented excessive mitochondrial iron uptake caused by the ferroptosis inducers. Further, in one Drp1 KO cell line, the resilience against ferroptosis was further enhanced through metabolic effects, i.e., reduced mitochondrial respiration and a compensation of ATP production through glycolysis.

We previously reported that Drp1 inhibition preserved cellular integrity in conditions of glutamate toxicity by preventing mitochondrial fragmentation and mitochondrial membrane depolarization [16]. The present study significantly extends our previous findings applying an erastin- and RSL3-mediated ferroptosis model in Drp1 knockout cell lines and now highlights the role of mitochondrial metabolism and iron homeostasis in these model systems of ferroptosis.

It is well established that inactivation of Drp1 function leads to the elongation of mitochondria [30, 31], whereas oxidative stress causes excessive mitochondrial fragmentation [25, 32]. Our results are in line with these earlier findings and, in addition, demonstrate that Drp1 deficiency inhibits mitochondrial fission caused by erastin, but not by RSL3 (Fig. 1C, D). However, mitochondria remain significantly elongated in the Drp1 KO cell lines also during RSL3 treatment when compared to the HT22 Drp1 expressing cell lines (Fig. 1C, D).

Based on the data obtained in this study in the models of ferroptosis, Drp1 induces mitochondrial fission within 6 h of ferroptosis-induction (Fig. 1C, D), and concomitantly, iron is taken up into the mitochondria (Fig. 2A, B). Previous studies have found that iron overload causes excessive mitochondrial fission, which is then mediated by Drp1 [33, 34]. How Drp1 deficiency and the associated mitochondrial elongation alters mitochondrial iron homeostasis, however, is still elusive. Our results show that Drp1 deficiency abrogated mitochondrial iron uptake that was caused by the ferroptosis inducers (Fig. 2A, B). Notably, erastin or RSL3-mediated mitochondrial iron uptake occurred between 2 and 6 h of treatment, preceding ferroptosis mediated decline of mitochondrial integrity in the neuronal HT22 cells. This time course of ferroptosis-induced mitochondrial fission is in line with findings in our previous studies [14, 25], suggesting that increased mitochondrial iron uptake and concomitant mitochondrial fission are upstream events, initiating the devastating damage of the mitochondria during ferroptosis. This is supported by data from the present study showing that deferoxamine, which is established to chelate iron and protect against ferroptosis, inhibited erastin- or RSL3-mediated mitochondrial iron uptake (Fig. 1SC), impairment of mitochondrial integrity and ultimately ferroptotic cell death (Fig. 6C, E).

Further, Drp1 deficiency led to increased cytosolic iron levels between 2 and 6 h of erastin and RSL3 treatment, which was not seen in the HT22 controls (Fig. 1SA, B). Several studies have shown the interconnection of mitochondria on iron metabolism through iron-sulfur clusters [35–37] that serve as binary switch on Iron Regulating Proteins (IRPs) [36]. IRPs and their binding to the Iron Responsive Element (IRE) tightly regulate protein expression of either iron storage proteins like ferritin or iron uptake proteins like the transferrin-receptor [36, 38, 39]. To which extent Drp1 deficiency or mitochondrial elongation influences iron-sulfur cluster assembly and the regulation of IRPs, is not clarified. Our data show increased cytosolic iron uptake up to 6 h within induction of ferroptosis in the Drp1 KO cell lines (Fig. 1SA, B). After 8 h however, cytosolic iron levels decline in erastin and RSL3 treated cells. These results suggest that Drp1 depletion might lead to the expression of iron uptake proteins, which was triggered by erastin and RSL3 treatment.

On the other hand, it is well established that excessive labile iron within mitochondria contributes to mitochondrial ROS production [40, 41], leading to devastating damage of mitochondrial function and integrity. Recent studies confirmed that mitochondrial lipid peroxidation was critical in ferroptosis by using a fluorescent mitochondria-targeted dye that is sensitive to lipid peroxidation [42]. Our results show that Drp1 deficiency prevented ferroptosis-mediated accumulation of mitochondrial ROS and mitochondrial lipid peroxidation (Fig. 2C–F). Furthermore, this protection from excessive mitochondrial ROS formation also preserved mitochondrial integrity in the Drp1 knockout cell lines as indicated by the measurements of the mitochondrial membrane potential.

Strikingly, our data show that mitochondrial and cellular integrity was preserved in the Drp1 knockout cell lines despite the depletion of GPX4 (Fig. 4C) and the accumulation of overall lipid peroxides (Fig. 4A, B). These findings suggest that preventing mitochondrial lipid peroxidation (Fig. 2E, F) and preserving mitochondrial integrity is far more important for cellular protection against ferroptosis than interfering with early features of ferroptosis such as GSH depletion, GPX4 inhibition or moderate lipid peroxidation. In fact, our data from the present study are in line with earlier findings [14, 16, 43] and impose Drp1 mediated mitochondrial damage as a key event in ferroptosis that amplifies the initiated oxidative stress and marks the “point of no return” in this form of oxidative cell death [25, 44–46].

In addition to Drp1-dependent mitochondrial fission, formation of mitochondrial hemi-fusion sites as membrane binding spots of pro-apoptotic BAX and BID, the attenuation of mitochondrial respiration, may a contribute into cellular resilience against oxidative stress through the shift of mitochondrial metabolism to glycolysis [14, 28, 43]. The same effect is seen in the Drp1 KO #1 cell line, but not in the Drp1 KO #2 cell line (Fig. 3D-G). The observed difference in effects on mitochondrial metabolism between the Drp1 KO cell lines were also correlated with other observations of this study, since the Drp1 KO #2 cell line did not completely abrogate erastin and RSL3-mediated accumulation of mitochondrial ROS and mitochondrial lipid peroxidation (Fig. 2C-F). In contrast, we observed that the basal and maximal mitochondrial respiration were significantly reduced in the Drp1 KO #1 cell line (Fig. 3D), and this cell line was far more resistant to increasing concentrations of erastin or RSL (Fig. 6A, B), showed stronger effects on mitochondrial ROS formation compared to the Drp1 KO #2 cell line (Fig. 2C, D), and also attenuated the overall lipid peroxidation upon ferroptosis induction (Fig. 4A, B). In contrast to our data obtained from neuronal cells, basal and maximal oxygen consumption was not significantly different from controls in *Drp1* mutant fibroblasts of patients with EMPF1, while these cells also showed an upregulation of glycolysis [47]. Apparently, the de novo missense mutations examined in these patients maintain the functionality of the Drp1 protein, thereby preserving mitochondrial respiration and leading to extended life expectancy into childhood or early adolescence. This differs from the existing knockout model, which, although tolerated in vitro, exhibits lethality in global *Drp1* knockout mouse models [19] and also mostly leads to lethality in humans [10, 48]. How Drp1 depletion affects energy metabolism is unknown and requires further investigation. Our findings suggest that the observed metabolic impact on mitochondrial respiration may significantly contribute further to the protective effects of Drp1 knockout on mitochondrial pathways of ferroptosis.

Overall, this study underlines the importance of mitochondrial metabolism and function in the ferroptosis pathway. Our results highlight the detrimental role of mitochondria to amplify ROS production under conditions of oxidative stress. Many studies accentuate mitochondria as the major source for cellular ROS [43, 49]. The present study shows that through preserving mitochondrial integrity during ferroptosis, the detrimental mitochondrial iron accumulation, ROS production, and ultimately cellular demise can be prevented despite erastin- or RSL3-mediated inhibition of GPX4 activity or accumulating lipid peroxidation. The involvement of mitochondria in the ferroptosis pathway is an essential mechanism of ROS amplification and further death signaling, and only in extreme conditions of ferroptosis, mitochondria lose their significance to prevent cell death [50]. In summary, this study leads to the conclusion that Drp1-mediated mitochondrial disintegration plays an essential role in the course of ferroptotic cell death.

## Materials and methods

### Cell culture

Immortalized mouse hippocampal HT22 cells were grown in Dulbecco’s modified Eagle medium (DMEM, Capricorn, Germany), supplemented with 10% heat-inactivated FCS, 10% Penicillin/Streptomycin and 2 mM glutamine. erastin and RSL3 were solved in DMSO and diluted in DMEM for the indicated concentrations prior to the induction of ferroptosis. The cells were incubated with respective ferroptosis inducers for the indicated time intervals.

### DNA transfection

Transfections were conducted using Attractene Transfection Reagent (Qiagen, Hilden, Germany) according to the manufacturer’s protocol. In short, DNA is pipetted into Serum-reduced Opti-MEM (Gibco, Thermo Fisher Scientific, Schwerte, Germany) with subsequent addition of Attractene. After 15 min of complex formation, the transfection reagents were pipetted dropwise to the cells.

### Generation of CRISPR/Cas9 knockout colonies

Drp1 CRISPR/Cas9 KO cell lines were generated from HT22 cells by the transient transfection of a plasmid (pSpCas9(BB)-2A-GFP, Addgene, MA, USA) containing the single guide RNA with the sequence addressing the GTPase Domain of the Drp1 protein (GGTCATGGAGGCGCTGATCC), a GFP reporter and the Cas9 nuclease, following fluorescence-activated single-cell sorting into 96-well-plates in the MoFlo Astrios Cell Sorter (Beckman Coulter GmbH, Krefeld, Germany).

### gDNA analysis

Genomic DNA of respective cell lines was extracted using Monarch Genomic DNA Purification Kit (New England Biolabs, MA, USA) according to the manufacturer’s protocol. The gDNA was used for PCR to amplify the region around the target sequence of the single guide RNA using primer up- and downstream of the cleavage site (fw: GTCTGCTCACAGCAACTCCT, rev: GTCTGAGCTTCCTTACCCGC). The PCR product was run through a gel electrophoresis and the specific band was excised from the gel and purified using Monarch DNA Gel Extraction Kit according to the manufacturer’s protocol. The resulting PCR product was prepared and sent to Eurofins Genomics Europe Shared Services GmbH (Ebersberg, Germany) for Sanger Sequencing.

### ATP-assay

ATP levels were assessed using ViaLightplus Bioassay Kit (Lonza, Verviers, Belgium) according to the manufacturer’s protocol. In short, 7 000 cells/well of respective colonies were seeded into 96-well plates. After induction of ferroptosis, a lysis buffer was added to lyse the cells. The cell lysates were then transferred to a luminescence plate (Greiner, Frickenhausen, Germany). An ATP monitoring reagent was added and incubated in the dark. Luminescence was measured using the FluoStar OPTIMA photometer (BMG Labtech, Offenbach, Germany).

### MTT-assay

Cells were seeded into 96-well plates (7 000 cells/well) and grown in the incubator at 37 °C and 5% CO_2_. After induction of ferroptosis, MTT-reagent (Sigma Aldrich, MO, USA) was added to the culture medium to a concentration of 2.5 mg/ml and incubated for an hour at 37 °C. The tetrazolium salts are solved in DMSO following absorbance measurement at 570 nm versus 630 nm with the FluoStar OPTIMA photometer (BMG Labtech, Offenbach, Germany).

### Annexin V/PI assay

To assess cell viability, cells were seeded into 24-well-plates at 40 000 cells/well. After induction of ferroptosis, the cells were trypsinized, centrifuged and resuspended in PBS containing 18 µg/well Annexin V-FITC (BioLegend, CA, USA) and 1:1000 propidiumiodide (Cayman Chemical, MI, USA) for 5 min in the dark at room temperature. The red and green fluorescence of 5000 cells were measured in an easyCyte flow cytometer (Luminex, TX, USA) (excitation 488 nm, emission red: 690/50 nm, green: 525/30 nm).

### BODIPY-C11/MitoperOx/TMRE/PhenGreen SK diacetate

Lipid peroxidation (BODIPY-C11), lipid peroxidation within mitochondria (MitoPerOx), mitochondrial membrane potential (TMRE) and cytosolic iron levels (PhenGreen SK Diacetate) were assessed using FACS analysis.

Cells were seeded into 24-well plates (40 000-60 000 cells/well). After the induction of ferroptotic cell death, the cells were stained with the specific solution (2 µM Bodipy, 100 nM MitoPerOx and 400 nM TMRE and 5 µM PhenGreen SK Diacetate) at 37 °C. After 30 min the cells were trypsinized, centrifuged and resuspended in PBS.

For BODIPY-C11 and PhenGreen SK Diacetate, fluorescence was excited at 488 nm wavelength and emission recorded at 525/30 nm and 690/50 nm wavelengths. MitoPerOx displays an excitation maximum of 495 nm and exhibits a shift in emission maxima from 590 to 520 nm upon mitochondrial lipid peroxidation, enabling determination of ratiometric measurements of lipid peroxidation in live cells. TMRE was measured at 488 nm excitation and 690/50 nm emission wavelength.

### MitoSox assay

MitoSOX was used for the detection of reactive oxygen species in mitochondria, therefore cells were seeded into 24-well plates at 40,000 cells/well.

For FACS analysis, after the indicated treatment, cells were harvested with trypsin after washing once with PBS. After induction of ferroptotic cell death, the cells were trypsinized, centrifuged, and resuspended in PBS containing a 1.25 µM MitoSOX solution and incubated for 30 min at 37 °C. MitoSOX measurements are conducted at an excitation wavelength of 488 nm and an emission of 690/50 nm.

### Seahorse XF analysis

To assess mitochondrial respiration and glycolytic activity, the Seahorse XFe Analysis Method was used in 96-well plates. During the measurement oligomycin, FCCP, a mix of rotenone and antimycin A, and 2-DG were successively injected to measure basal and ATP-linked respiration, maximal respiration, and non-mitochondrial respiration.

After ferroptotic cell death induction, culture medium was washed once with 100 μL and then replaced with 180 μL of Seahorse assay medium (DMEM containing 4.5 g/L glucose,2 mM L-Alanyl-L-glutamine, 1 mM Na-pyruvate, pH 7.35) and kept at 37 °C for 1 h. After calibration of the cartridge three baseline OCR/ECAR measurements were recorded before successively injecting port A-D (oligomycin (3 µM), FCCP (0.5 µM), a mix of rotenone (100 nM) and antimycin A (1 µM) and 2-DG (50 mM)) followed by three measurements.

### Assessment of mitochondrial morphology

To assess mitochondrial morphology, 17,000 cells have been plated into Ibidi 8-well slides and treated with ferroptosis inducers for the indicated time intervals. Prior to the measurement, the cells were stained with 200 nM MitoTracker Deep Red FM (Invitrogen, Thermo Fisher Scientific, Schwerte, Germany) solved in DMSO and diluted in DMEM for half an hour at 37 °C. The staining solution was discarded and PBS was added to the cells. Measurements were conducted in a Leica DM6000 epi-fluorescence microscope (Wetzlar, Germany) (60x immersion oil objective) (excitation: 630/60 nm, emission: 700/75 nm).

Mitochondrial Morphology was analyzed using an ImageJ Plugin as described in ref. [51].

### Mito-FerroGreen assay

Mito-FerroGreen (Dojindo EU GmbH, Munich, Germany) detects ferrous iron in mitochondria. For this, 17 000 cells were seeded into Ibidi-8-wells. Ferroptosis was induced for the indicated time intervals. Prior to the measurement, cells were washed with 1x PBS and stained with 5 µM of Mito-FerroGreen solved in DMSO and diluted in 1× PBS for 15 min. The staining solution was discarded and the cells were measured in PBS in a Leica DM6000 epi-fluorescence microscope (Wetzlar, Germany) (20× objective) (excitation: 488 nm, emission: 525/30 nm).

The fluorescence was measured using ImageJ by placing ROIs around each cell and calculated by subtracting respective background fluorescence from the cell fluorescence.

### Western blot

For the determination of protein levels, 200 000-250 000 cells were seeded into 6-well-plates. After respective treatments, cells were washed once with 1x PBS and lysed in Lysis Buffer, that contains of 0.05 M Tris, 1 M EDTA, 1 M EGTA, 0.25 M Mannitol, 1 mM DTT, 0.25% Triton-X-100, PhosSTOP (Roche, Basel, Switzerland) and cOmplete ULTRA Tablets Protease Inhibitor (Roche, Basel, Switzerland). The soluble protein fraction is extracted from the supernatant after centrifuging at 10,000 *g* for 10 min at 4 °C. The total amount of protein is determined by using the Pierce BCA Protein Assay Kit (Thermo Fisher Scientific, Schwerte, Germany). 35 µg of respective protein conditions are loaded into 12.5% gels and blotted onto a PVDF-membrane at 325 mAh for 2–3 h. After blocking in 5% skim milk, respective primary antibodies ms-Drp1 (1:1000, BD Biosciences, Franklin Lakes, NJ, USA), rb-GPX4 (1:250, Thermo Fisher Scientific, Schwerte, Germany), ms-Vinculin (1:100,000, Sigma-Aldrich, St. Louis, MO, USA) and goat-GFP (1:500, Biomol GmbH, Hamburg, Germany) were incubated overnight following 1 h of incubation with the corresponding secondary antibody (goat anti-rabbit 1:2500, Vector-Laboratories, Newark, CA, USA), goat anti-mouse 1:3000, Vector-Laboratories, Newark, CA, USA), which is coupled with an HRP. The proteins were detected via chemiluminescence using the ChemiDoc (Bio-Rad, Feldkirchen, Germany). The analysis was conducted using the ImageLab Software (Bio-Rad, Feldkirchen, Germany).

### Supplementary information

Supplementary Material

## Data Availability

The original datasets and analyses of the current study are available from the corresponding author on reasonable request.
