# Supplementary material for: Drp1 depletion protects against ferroptotic cell death by preserving mitochondrial integrity and redox homeostasis
Source: Cell Death Dis. 2024 Aug 27;15(8):626. doi: 10.1038/s41419-024-07015-8 (PMC11350090; doi:10.1038/s41419-024-07015-8)
Supplement: Supplementary file 1 — Supplementary Material [file 41419_2024_7015_MOESM1_ESM.docx]

**Drp1 depletion protects against ferroptotic cell death by preserving mitochondrial integrity and redox homeostasis**

Stephan Tang*^1,2^, Anneke Fuß*^1,2,3^, Zohreh Fattahi^1,2^, Carsten Culmsee^1,2^

^1^ Institute for Pharmacology and Clinical Pharmacy, Philipps-University Marburg, Karl-von-Frisch-Str. 2, 35043 Marburg, Germany

^2^ Marburg Center of Mind, Brain, and Behaviour – CMBB, Hans-Meerwein-Straße 6, 35032 Marburg, Germany

^3^ Institute of Reconstructive Neurobiology, Neurodevelopmental Genetics, University Bonn, LIFE & BRAIN Center, Sigmund-Freud-Str. 25, 53127 Bonn, Germany

* These authors contributed equally to this work

Corresponding Author: Prof. Dr. Carsten Culmsee, Karl-von-Frisch-Str. 2, 35043 Marburg, Germany, +49-(0)6421-2825780, Carsten.culmsee@staff.uni-marburg.de

**

**

**Figure 1S. Iron transmission is altered during Drp1 deficiency and the chelation of iron by Deferoxamine**

**A, B** Cytosolic Iron content was assessed using PhenGreen SK Diacetate, which is quenched upon iron binding. Drp1 KO cells show increased cytosolic uptake of iron between 2-6 hours of 0.5 µM erastin and 100 nM RSL3 treatment and a decrease of cytosolic iron after 8 hours. Values are shown as mean ± SD; 5000 cells per replicate of n = 3 replicates. ns non-significant, ** p<0.01, *** p<0.001 compared to untreated control (One Way ANOVA, Bonferroni’s post-hoc Test). **C** Deferoxamine abolishes erastin and RSL3 mediated mitochondrial iron uptake. HT22 cells were treated with 0.5 µM erastin and 100 nM RSL3 and respectively co-treated with 10 µM deferoxamine for the indicated time points. The individual values are depicted as an arbitrary fluorescence value; n = 100-125 replicates; ns non-significant, *** p<0.001 compared to respective untreated control, ### p<0.001 in comparison to respective time point of the treated control (One Way ANOVA, Bonferroni’s post-hoc Test).

**
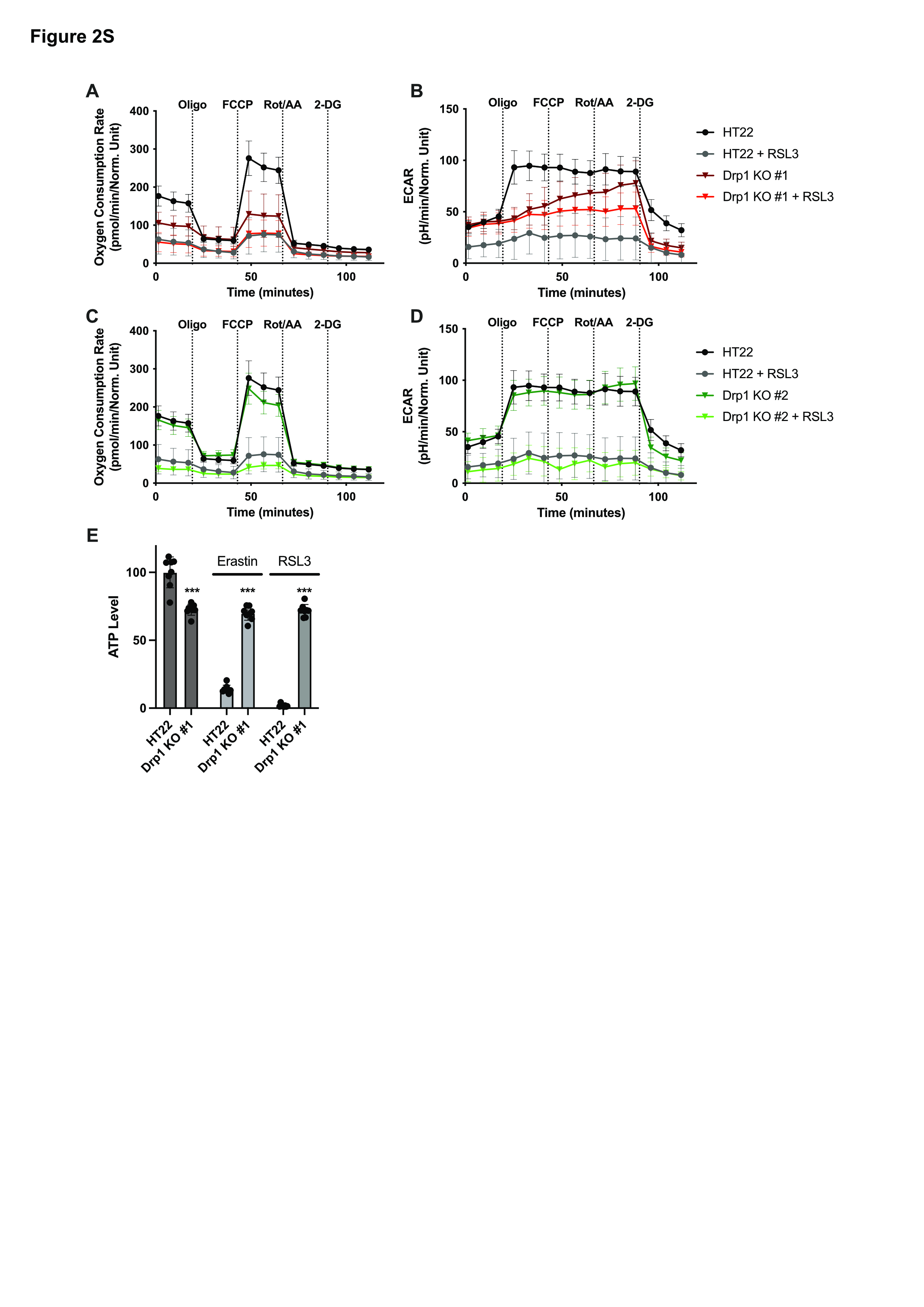
**

**Figure 2S. Drp1 deficiency balances ferroptosis mediated loss of ATP level but not RSL3-mediated impairment of mitochondrial respiration and glycolytic activity**

**A-D** The oxygen consumption rate and the extracellular acidification rate was measured using the Seahorse XF96-Analyzer. The cells were treated with 100 nM RSL3 for 16 hours and measured. The oxygen consumption rate and the extracellular acidification rate were normalized to respective protein content. The traces are depicted as mean ± SD at n=6-8.

**

**

**

**

**

**
